# Study and Modification of the Polycyclic Aromatic Hydrocarbon Degradation Gene Cluster in *Burkholderia* sp. FM-2

**DOI:** 10.3390/microorganisms13092079

**Published:** 2025-09-06

**Authors:** Jiajun Ma, Ying Zhai, Yumeng Cui, Guohui Gao, Ming Ying, Yihe Zhao, Agostinho Antunes, Lei Huang, Meitong Li

**Affiliations:** 1 Tianjin Key Laboratory of Organic Solar Cells and Photochemical Conversion, College of Chemistry and Chemical Engineering, Tianjin University of Technology, Tianjin 300384, China; 2 CIIMAR/CIMAR, Interdisciplinary Centre of Marine and Environmental Research, University of Porto, Terminal de Cruzeiros do Porto de Leixões, Av. General Norton de Matos, s/n, 4450-208 Porto, Portugal; 3 Department of Biology, Faculty of Sciences, University of Porto, Rua do Campo Alegre, s/n, 4169-007 Porto, Portugal

**Keywords:** polycyclic aromatic hydrocarbons, bioremediation, transcriptomics, bioinformatics, salicylate hydroxylase

## Abstract

Polycyclic aromatic hydrocarbons (PAHs) are a class of persistent organic pollutants composed of two or more fused benzene rings, posing serious threats to ecological environments and human health. Biodegradation is an efficient, economical, and sustainable approach for remediating PAHs pollution. In our previous work, we isolated and characterized a PAH-degrading bacterium, *Burkholderia* sp. FM-2. FM-2 demonstrated strong tolerance and efficient degradation capacity toward various PAHs, achieving 81.98% degradation of 2 mM phenanthrene within 3 days, and over 58% degradation of 2 mM fluorene, dibenzofuran, and dibenzothiophene under the same conditions. Through combined genomic and transcriptomic analyses, a putative PAH degradation gene cluster was identified in the FM-2 genome. Phylogenetic and domain architecture analyses were conducted on seven oxygenase genes within the cluster. Using AlphaFold 3, we predicted the three-dimensional structure of the downstream transport protein OmpW and proposed a potential transmembrane channel for PAHs uptake. To eliminate the phenanthrene degradation intermediate 1-hydroxy-2-naphthoic acid, a genetically engineered strain FM-2::*nahG* was constructed by heterologous expression of the salicylate hydroxylase gene (*nahG*). The modified strain completely abolished the accumulation of 1-hydroxy-2-naphthoic acid and achieved complete mineralization of phenanthrene. This study not only reveals the molecular basis of PAHs degradation in *Burkholderia* sp. FM-2 but also demonstrates the potential of metabolic engineering to enhance biodegradation ability, providing a promising microbial candidate for the bioremediation of PAH-polluted environments.

## 1. Introduction

Polycyclic aromatic hydrocarbons (PAHs) are organic compounds characterized by the presence of two or more fused benzene rings. The majority of these substances are colorless solids, exhibiting hydrophobicity, resistance to degradation, and persistence, which contribute to their stable existence in the environment [1]. Primary natural sources of PAHs include forest fires, volcanic eruptions, and emissions from biological processes in plants and bacteria. Additionally, anthropogenic activities such as fossil fuel combustion and processing, creosote production, and petroleum extraction and refining of petroleum have been identified as significant contributors to PAHs pollution [2]. Long-term accumulation of PAHs has been demonstrated to increase the risk of carcinogenesis, teratogenesis, and mutagenesis in humans. Furthermore, they have the capacity to modify the composition of microbial communities and to diminish environmental biodiversity, a phenomenon that has the potential to result in severe ecological disasters [3].

A plethora of remediation strategies for PAHs pollution have been identified, which can be categorized into three distinct methods: physical, chemical, and biological. Bioremediation is a more efficient, environmentally friendly, and cost-effective approach. The bioremediation process utilizes the metabolic capabilities of plants and microorganisms to transform, reduce, or eliminate pollutants from the environment. Many PAH-degrading bacteria have been reported, including *Burkholderia*, *Mycobacterium*, *Streptomyces*, and others [4,5,6]. The degradation of PAHs by bacteria principally occurs via aerobic and anaerobic pathways. The initial step of anaerobic degradation primarily relies on carboxylase to catalyze the carboxylation of the aromatic ring, whereas aerobic degradation mainly depends on various oxygenases [7,8]. To illustrate this process, consider the aerobic degradation of phenanthrene. Initially, phenanthrene is hydroxylated by oxygenases, resulting in the formation of 1,2-phenanthrenediol or 3,4-phenanthrenediol. Subsequent ring cleavage then leads to the formation of two-ring intermediates, namely 1-hydroxy-2-naphthoic acid or 2-hydroxy-1-naphthoic acid [9]. 1-Hydroxy-2-naphthoic acid can undergo further hydroxylation, resulting in the naphthalene degradation pathway, or direct ring opening, leading to the phthalic acid pathway. In the naphthalene degradation pathway, the two-ring compound is further cleaved into the single-ring compound salicylic acid. Salicylic acid can then enter either the catechol pathway or the gentisic acid pathway, ultimately entering the tricarboxylic acid (TCA) cycle to achieve complete mineralization.

In our laboratory’s previous work, an efficient PAH-degrading bacterium, *Burkholderia* sp. FM-2, was identified, exhibiting high degradation capability for both naphthalene and phenanthrene [10]. However, in the prior study, the genetic pathway underlying PAHs degradation by FM-2 had not been elucidated. This study further evaluated the degradation capacity of FM-2 towards a broader range of PAHs. Through techniques such as genome sequencing and transcriptomic analysis, the gene cluster involved in PAHs degradation was identified and characterized. Furthermore, a detailed investigation into the oxygenase genes and transporter genes within this cluster was conducted. Finally, this study is the first to report the complete degradation of the intermediate 1-hydroxy-2-naphthoic acid by introducing a heterologous salicylate hydroxylase gene.

## 2. Materials and Methods

### 2.1. Bacteria, Chemicals and Culture Media

The *Burkholderia* sp. FM-2 was isolated from petroleum-contaminated soil samples collected from the oilfield in Xinjiang, China, in a previous study conducted by our laboratory [10]. Phenanthrene (97% purity), fluorene (99% purity), dibenzofuran (98% purity), dibenzothiophene (99% purity) and pyrene (97% purity) was procured from Shanghai Macklin Biochemical Co., Ltd. (Shanghai, China), ethyl acetate and methanol (chromatographic-grade) from Tianjin Concord Science and Technology Co., Ltd. (Tianjin, China). All other Chemicals were procured from Tianjin Damao Chemicals Reagent Factory (Tianjin, China). Mineral salt medium (pH 7 ± 0.2) containing 0.7 g MgSO_4_, 3.48 g KH_2_PO_4_, 1.5 g Na_2_HPO_4_·12H_2_O, 3.96 g (NH_4_)_2_SO_4_, and 0.01 g yeast per L of distilled water.

### 2.2. Determination of PAHs Degradation Rate

PAHs were dissolved in ethyl acetate to prepare a 200 mM stock solution, which was added to the medium and then allowed to stand for complete evaporation of ethyl acetate. FM-2 was cultured at 25 °C, 200 rpm to determine the degradation rate of PAHs. The absorbance at 600 nm (OD600) (Shanghai Spectrum Instruments Co., Ltd., Shanghai, China) was employed to monitor FM-2 growth. The culture solution was extracted three times with equal volumes of ethyl acetate. The obtained extract was evaporated under reduced pressure, and the residue was dissolved in chromatographic-grade ethyl acetate and adjusted to a uniform volume. The concentration of PAHs was determined using a gas chromatograph (GC) (GC9790 Plus, Fuli Instruments, Zhejiang, China) with the following parameters: injector temperature, 300 °C; detector temperature, 330 °C; capillary column, 50 m × 0.25 mm × 0.25 μm; oven temperature program: held at 130 °C for 3 min, then increased at a rate of 45 °C/min to 180 °C and held for 5 min, subsequently increased at a rate of 45 °C/min to 280 °C and held for 5 min; injection volume, 1 μL.

### 2.3. Genome Sequencing and Assembly

Genomic DNA was extracted from bacterial cells using Bacterial/fungal DNA extraction kit (magnetic beads) (Majorbio, Shanghai, China). After quality assessment of the DNA by using NanoDrop 2000 (Thermo Fisher Scientific, Waltham, MA, USA), the samples were fragmented by ultrasonication. The purified DNA fragments were then used for library construction and subsequent sequencing [11,12]. Briefly, DNA samples were sheared into ~400 bp fragments using a Covaris M220 Focused Acoustic Shearer following manufacture’s protocol. Illumina sequencing libraries were prepared from the sheared fragments using the NEXTFLEX Rapid DNA-Seq Kit (Bioo Scientific, Austin, TX, USA). 5′primer ends were first end-repaired and phosphorylated. Next, the 3′ ends were A-tailed and ligated to sequencing adapters. The third step is to enrich the adapters-ligated products using PCR. The prepared libraries then were used for paired-end Illumina sequencing (2 × 150 bp) on Illumina Novaseq 6000 (Illumina Inc., San Diego, CA, USA). Raw reads obtained after sequencing were filtered using a fastp software (version 0.19.6) followed by assembly with SOPA de novo version 2.04 [11,13]. Glimmer was used for CDS prediction, tRNA-scan-SE was used for tRNA prediction and Barrnap was used for rRNA prediction [14,15]. The predicted CDSs were annotated from NR, Swiss-Prot, Pfam, GO, COG, KEGG and CAZY database using sequence alignment tools such as BLASTP, Diamond and HMMER.

### 2.4. RNA Extraction, Transcriptome Assembly and Analysis

Phenanthrene were dissolved in ethyl acetate to prepare a 200 mM stock solution. Strain FM-2 was inoculated into mineral salt medium supplemented with 2 mM phenanthrene, and a control culture was inoculated into medium containing 1% glucose without phenanthrene. There were three replicates for both the experimental group and the control group. Total RNA was extracted using an RNAprep Pure Cell/Bacteria Kit (Tiangen, Beijing, China), according to manufacturer’s instructions. The quality of RNA was assessed using agarose gel electrophoresis, NanoDrop 2000 and Fragment Analyzer 5300 (Agilent, Santa Clara, CA, USA). The transcriptomic sequencing was performed by Majorbio (Shanghai, China) using the NovaSeq X Plus platform (Illumina Inc., San Diego, CA, USA). The “DESeq2” package in R software was applied to screen DEGs [16]. The differential genes DEGs were transmitted into GO (gene ontology) and KEGG (Kyoto Encyclopedia of Genes and Genomes) analyses. The *p*-values were corr I removed it. ected using the Benjamini–Hochberg (BH) method. Genes with a *p*-value < 0.05 and |log2FC| > 1 were classified as differentially expressed.

### 2.5. Validation of RT-qPCR

RT-qPCR was performed using SYBR green fluorescent dye (GenStar, Beijing, China) on a two-color real-time PCR detection system (LightCycler^®^ 96, Roche, Switzerland). The experimental data were analyzed according to the 2^−ΔΔCt^ method, and standardized by the 16S rRNA gene as an internal reference control. The primer sequences used in the experiments are listed in Table S1 of the Supplementary Information.

### 2.6. Bioinformatics Analysis

Protein sequence alignment and phylogenetic analysis were performed using Mega 11.0 software, and visualization was created using ESPript 3.0.

The domain analysis of oxygenases was conducted using HMMER and InterPro [17,18].

The structure of the transporter OmpW was predicted using AlphaFold 3 [19].

The hydrophobicity analysis of the transporter OmpW was performed using Discovery Studio 2019.

The transport channel of phenanthrene within the transporter OmpW was predicted using CAVER Analyst 2.0 [20].

### 2.7. Detection of Phenanthrene Degradation Intermediates

FM-2 was inoculated into 50 mL of mineral salt medium containing 2 mM phenanthrene and cultured at 25 °C, 200 rpm for 3 days. The culture medium was extracted using the same method described in Section 2.2. After evaporation under reduced pressure, the residue was dissolved in 3 mL chromatographic-grade methanol. The samples were analyzed by high-performance liquid chromatography coupled with mass spectrometry (HPLC-MS) (Xevo G2 Q-Tof, Waters, Milford, MA, USA). The mobile phase consisted of methanol and water at a ratio of 70:30, with a flow rate of 0.5 mL/min. Detection was performed at a wavelength of 254.0 nm.

### 2.8. Construction and Electroporation of the nahG Expression Vector

The *nahG* gene was codon-optimized and synthesized by Genewiz (Jiangsu, China), and its sequence information is provided in Supplementary Information. The completed plasmid was introduced into FM-2 cells using an electroporator (MicroPulser, Bio-Rad, Hercules, CA, USA). The steps for electroporation were as follows: mix 50 μL of cell suspension with 1–5 μg of plasmid DNA in a pre-cooled electroporation cuvette, apply a 2.0 kV electric pulse, and immediately add 1 mL of LB medium to recover the cells for 1 h. The cell suspension was then centrifuged at 5000 rpm for 1 min, resuspended in ddH_2_O, and spread onto LB agar plates supplemented with kanamycin. The plates were incubated at 25 °C for 3 days. Colonies that were successfully transformed were screened and verified by PCR.

## 3. Results

### 3.1. Degradation of PAHs by Burkholderia sp. FM-2

**Figure 1:**
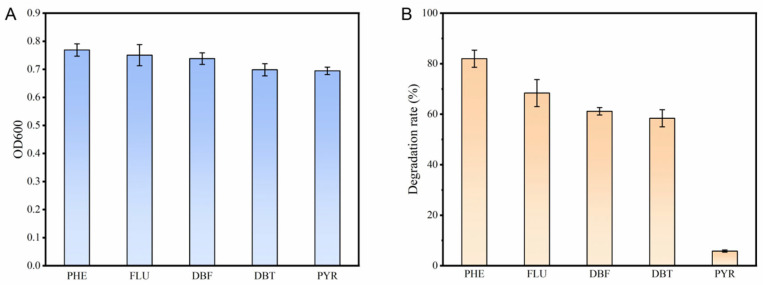
(**A**): Growth of FM-2 in the presence of different PAHs (2 mM); (**B**): Degradation rates of different PAHs (2 mM) by FM-2.

It has been demonstrated that *Burkholderia* sp. FM-2 exhibits good degradation capability towards various polycyclic aromatic hydrocarbons (PAHs). Strain FM-2 was inoculated into media supplemented with different PAHs (2 mM) and incubated for 3 days. The results demonstrated that FM-2 possesses strong tolerance to multiple PAHs (Figure 1A). After 3 days of incubation, the culture media containing phenanthrene (PHE), fluorene (FLU), dibenzofuran (DBF), and dibenzothiophene (DBT) exhibited color changes, which were presumed to result from the formation of colored intermediate metabolites during the degradation process (Supplementary Information, Figure S1). Similar color changes in the culture medium during phenanthrene degradation were also observed in *Marinobacter* sp. N4 as reported by Wang et al. [21]. The culture medium supplemented with pyrene (PYR) did not exhibit any color change. The degradation capability of strain FM-2 towards different PAHs (2 mM) was further evaluated (Figure 1B). Following a three-day incubation period, FM-2 showed the highest degradation efficiency for phenanthrene, achieving a degradation rate of 81.98%. The degradation rates for 2 mM fluorene, dibenzofuran, and dibenzothiophene were 68.37%, 61.13%, and 58.37%, respectively. In contrast, FM-2 exhibited almost no degradation activity towards pyrene. The reason for this phenomenon is related to the pocket size of the active site of the enzyme catalyzing the initial steps of PAHs degradation, as well as the affinity between amino acid residues and the substrate [22]. For example, as reported by Kweon et al., the NidAB and NidA3B3 systems exhibit the largest substrate-binding pocket in their active sites, showing high degradation efficiency toward pyrene and fluorene, but lower activity toward other low-molecular-weight PAHs [23].

### 3.2. Genome Sequencing of Burkholderia sp. FM-2

**Figure 2:**
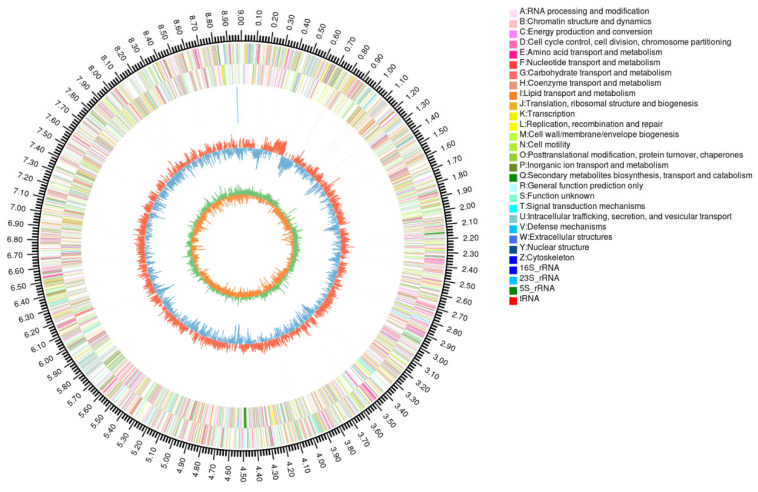
Circular representation of genome and features of *Burkholderia* sp. FM-2.

Strain FM-2 was subjected to whole-genome sequencing. The sequencing generated a total of 1,533,899,676 bp of raw data. After quality filtering, the dataset comprised 1,485,802,011 bp, with Q20 and Q30 quality scores of 98.39% and 94.71%, respectively. The assembly resulted in 112 scaffolds, with the longest scaffold measuring 1,011,379 bp (Figure 2). Genome assembly using dedicated software yielded a genome size of 9,009,556 bp, containing 9368 predicted coding sequences (CDS), with a GC content of 61.79%. The genome also contained 178 tandem repeats. Additionally, 60 tRNA genes, 2 rRNA genes (including one 16S rRNA and one 23S rRNA gene), and no 5S rRNA genes were identified. The genome sequence data of FM-2 have been deposited in the NCBI database under the accession number JAHNIZ000000000.1. Comparative genomic analysis identified a total of 93 oxygenase genes within the FM-2 genome, including 30 monooxygenase genes and 50 dioxygenase genes. Oxygenases play a crucial role in the aerobic degradation of PAHs [24]. The presence of a high number of oxygenase genes in the FM-2 genome is likely to be responsible for its ability to tolerate and degrade a variety of environmental pollutants. In the aerobic degradation pathway of PAHs, molecular oxygen is incorporated by oxygenases to activate the substrate, forming hydroxylated intermediate metabolites, which are subsequently cleaved via further oxygenase-catalyzed reactions [25].

### 3.3. Transcriptome Sequences Assembly and Analysis

**Figure 3:**
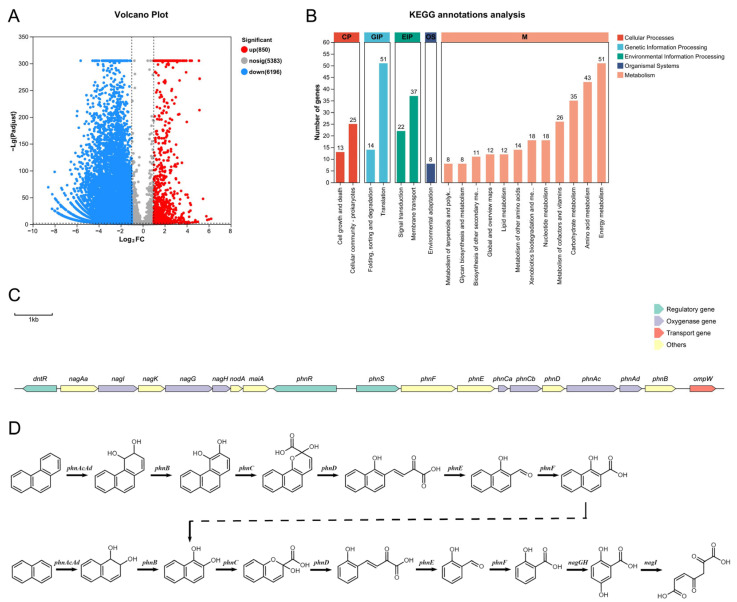
(**A**): Volcano Plot; (**B**): KEGG annotations analysis; (**C**): The gene clusters of PAHs degradation; (**D**): Proposed degradation pathways of phenanthrene and naphthalene in FM-2.

Transcriptomic analysis of strain FM-2 was performed. Compared to growth on glucose as the carbon source, a total of 850 differentially expressed genes (DEGs) were upregulated when FM-2 was grown on phenanthrene as the sole carbon source, accounting for 6.83% of the predicted genes in FM-2 (Figure 3A). KEGG annotation analysis revealed that the upregulated DEGs were primarily enriched in pathways related to energy metabolism, translation, amino acid metabolism, membrane transport, and carbohydrate metabolism (Figure 3B). Notably, 18 upregulated DEGs were identified in xenobiotics biodegradation and metabolism, which are potentially associated with PAH degradation. It is evident from the analysis of the data that, upon the identification of DEGs that have been expressed at elevated levels, a gene cluster that is implicated in the process of PAH degradation has been identified (Figure 3C). This gene cluster showed high similarity to the PAH degradation gene clusters reported in *Burkholderia* sp. strain RP007 by Laurie et al. and in *Burkholderia* sp. C3 by Tittabutr et al. [4,9]. The gene cluster contains three genes encoding transcriptional regulators: *dntR*, *phnR*, and *phnS*. It also includes seven genes encoding oxygenases: *phnAc* and *phnAd* (encoding PAH dioxygenase), *nagG* and *nagH* (encoding salicylate 5-hydroxylase), *phnCa* and *phnCb* (encoding aromatic ring-opening dioxygenase), and *nagI* (encoding gentisate 1,2-dioxygenase). Compared with previous reports, we additionally identified the gene *nodA*, encoding a non-heme iron oxygenase ferredoxin subunit; *maiA*, encoding maleylacetoacetate isomerase; and *phnCa*, encoding the LigA subunit of an aromatic ring-opening dioxygenase. In the downstream region of the gene cluster, at a distance of 377 base pairs, the *ompW* gene was identified. This gene encodes an outer membrane transporter protein, which may play a role in the transportation of polycyclic aromatic hydrocarbons. Genes encoding 1-hydroxy-2-naphthoate dioxygenase or salicylate hydroxylase were not detected in the genome of FM-2, implying that the strain may lack the capacity to metabolize the phenanthrene degradation intermediate 1-hydroxy-2-naphthoic acid. It is evident that, upon the identification of the relevant genes and consideration of the preceding study, a degradation pathway for phenanthrene and naphthalene in strain FM-2 was proposed (Figure 3D).

### 3.4. RT-qPCR Analysis

**Figure 4:**
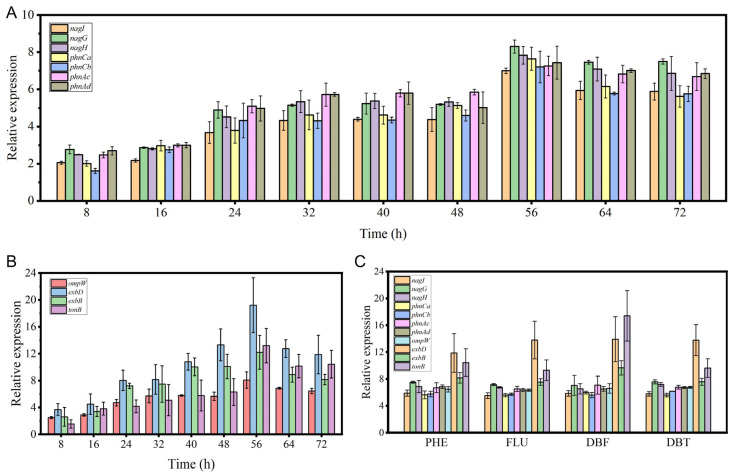
The results of RT-qPCR analysis. (**A**): Expression levels of oxygenase genes over time after addition of 2 mM phenanthrene; (**B**): Expression levels of transport genes over time after addition of 2 mM phenanthrene; (**C**): Expression levels of oxygenase and transport genes after 3 days of cultivation with 2 mM different PAHs.

The expression of oxygenase genes and transporter genes involved in PAH degradation by strain FM-2 was further validated using RT-qPCR. Upon addition of 2 mM phenanthrene to the medium, the transcription levels of oxygenase genes within the identified gene cluster gradually increased over time, reaching their maximum at approximately 56 h, with expression levels elevated more than 7-fold (Figure 4A). Among these, *nagG* exhibited the highest upregulation, increasing by 8.31-fold. The outer membrane transporter gene *ompW* and the TonB system-related genes *exbD*, *exbB*, and *tonB* showed a similar expression pattern, also peaking at 56 h (Figure 4B). The *exbD* gene displayed the greatest upregulation, reaching 19.22-fold. The subsequent decline in the expression of oxygenase and transporter genes after 56 h may be attributed to the decreasing substrate concentration as degradation progresses. Additionally, the expression levels of relevant oxygenase and transporter genes were examined after supplementation with different PAHs (2 mM) (Figure 4C). Following 3 days of incubation, the expression of these genes was significantly upregulated, indicating that this gene cluster plays a role in the degradation of various PAHs.

### 3.5. Dioxygenase of PAHs Degradation Pathway Analysis

**Figure 5:**
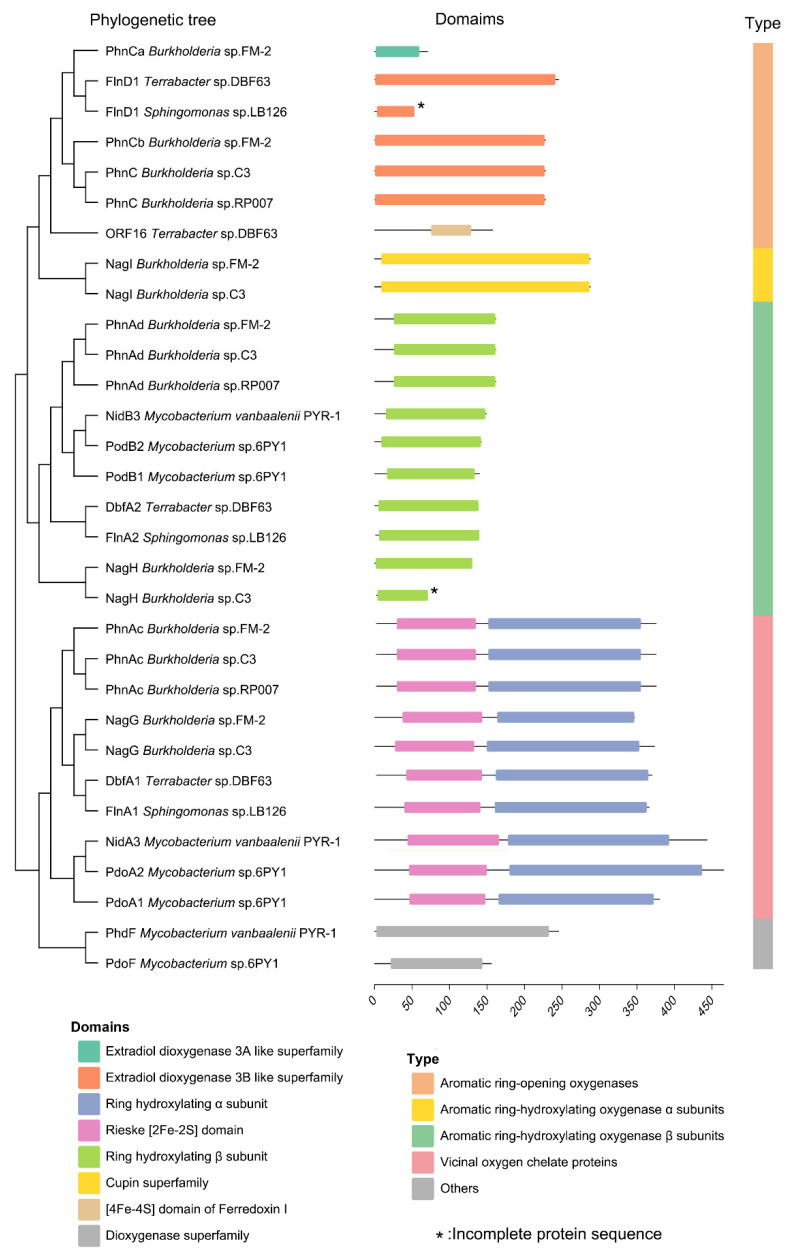
Phylogenetic tree and domain analysis of oxygenases involved in PAH degradation.

In the PAH degradation pathway of strain FM-2, three types of dioxygenases and one monooxygenase are involved. The PAH dioxygenase, encoded by *phnAc* and *phnAd*, catalyzes the initial step of PAH degradation by incorporating two adjacent hydroxyl groups onto the PAH molecule. The extradiol dioxygenase, encoded by *phnCa* and *phnCb*, catalyzes the cleavage of the extradiol ring, leading to ring opening of the PAH structure. The salicylate 5-hydroxylase, a monooxygenase encoded by *nagG* and *nagH*, is responsible for the conversion of salicylate, an intermediate in PAH degradation, to gentisate. Finally, gentisate 1,2-dioxygenase, encoded by *nagI*, catalyzes the further degradation of gentisate. Highly conserved gene clusters involved in PAH catabolism have been identified in numerous studies, and various PAH degradation genes have been reported, such as *no*, *pah*, *nid*, *dox*, *phn*, *phd*, *nag*, *fln*, etc. In this study, we selected the *phn* and *nag* gene clusters from *Burkholderia* sp. C3 and *Burkholderia* sp. RP007, which are reported to degrade naphthalene and phenanthrene [4,9]; the *nid* and *phd* gene clusters from *Mycobacterium vanbaalenii* PYR-1, reported to degrade naphthalene, phenanthrene, anthracene, fluoranthene, pyrene, and benz[a]anthracene [5]; the *pdo* gene cluster from *Mycobacterium* sp. strain 6PY1, reported to degrade pyrene [26]; the *fln* gene cluster from *Sphingomonas* sp. LB126, reported to degrade fluorene [27]; and the *fln* and *dbf* gene clusters from *Terrabacter* sp. DBF63, reported to degrade dibenzofuran [28]. These were used for sequence alignment and phylogenetic analysis of the dioxygenases within the PAH degradation gene cluster of strain FM-2 (Figure 5). Based on the resulting phylogenetic tree, these oxygenases can be broadly classified into four groups: aromatic ring-opening oxygenases, aromatic ring-hydroxylating oxygenase alpha subunits, aromatic ring-hydroxylating oxygenase beta subunits, and vicinal oxygen chelate (VOC) proteins. The extradiol dioxygenase and gentisate 1,2-dioxygenase from FM-2 both belong to the aromatic ring-opening oxygenase group, catalyzing the ring-cleavage step of aromatic compounds. The PAH dioxygenase and salicylate 5-hydroxylase belong to the aromatic ring-hydroxylating oxygenase group, responsible for the hydroxylation of the aromatic ring. Although PAH dioxygenase and salicylate 5-hydroxylase are classified as dioxygenase and monooxygenase, respectively, they are relatively conserved in both structure and sequence. The aromatic ring-hydroxylating dioxygenase alpha subunits from these species including FM-2 are composed of two domains: an N-terminal Rieske domain and a C-terminal catalytic domain. The Rieske domain contains a [2Fe-2S] cluster, where one Fe ion is coordinated by two conserved cysteine residues, and the other Fe ion is coordinated by two conserved histidine residues [29]. The Rieske domain functions in electron transfer during the catalytic process. The catalytic domain is the catalytic moiety of the aromatic ring-hydroxylating dioxygenase system, and its active site contains a non-heme ferrous ion coordinated by three ligands [30].

### 3.6. PAHs Transporter Analysis

**Figure 6:**
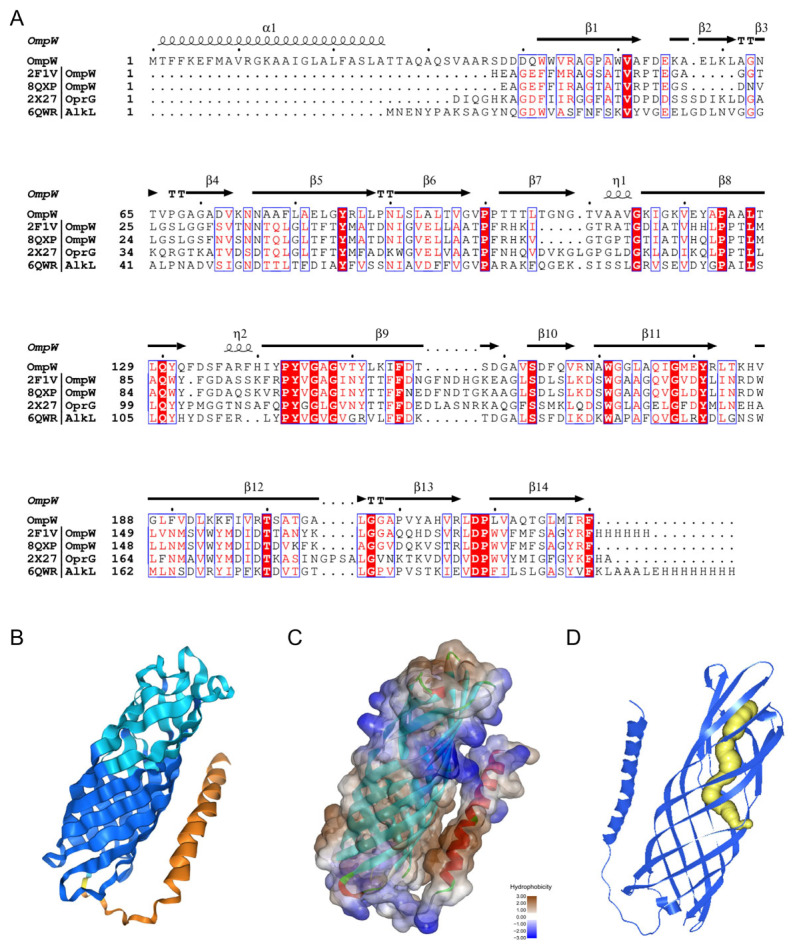
Structural analysis of the transporter OmpW. (**A**): Sequence alignment analysis with other OmpW family proteins; (**B**): Protein structure predicted by AlphaFold3; (**C**): Protein surface hydrophobicity analysis; (**D**): Predicted PAH molecule channel (The yellow portion).

A gene encoding an outer membrane transporter protein, *ompW*, was identified 377 bp downstream of the PAH degradation gene cluster in FM-2, which may be associated with the transport of polycyclic aromatic hydrocarbons (PAHs). OmpW is a β-barrel transmembrane protein; the hydrophobic interior of its β-barrel structure enables it to mediate the transmembrane transport of small hydrophobic molecules [31]. The amino acid sequence of OmpW from FM-2 was aligned with four representative OmpW family proteins. The sequence similarity between FM-2 OmpW and the OmpW from *Escherichia coli* K12 was 47.5%, and with that from *Klebsiella pneumoniae* was 43.7% (Figure 6A). The three-dimensional structure of OmpW was predicted using AlphaFold3 (Figure 6B), and its hydrophobicity was analyzed (Figure 6C). The predicted structure reveals an 8-stranded β-barrel conformation. The Grand Average of Hydropathicity (GRAVY) index for the entire OmpW protein is 0.263, indicating that it is a hydrophobic protein, which may facilitate the passage of polycyclic aromatic hydrocarbons (PAHs). The interior of the OmpW β-barrel forms a hydrophobic channel; however, the bottom of this channel exhibits hydrophilic characteristics (Supplementary Information, Figure S2). Therefore, PAH molecules are likely to diffuse laterally through the hydrophobic channel into the outer membrane (Figure 6D). This proposed transport mechanism is consistent with those reported for OmpW family proteins by Bert et al. and Hearn et al. [32,33]. Based on transcriptomic and RT-qPCR analyses, the expression levels of TonB-dependent receptor (TBDT)-related genes, including *exbD*, *exbB*, and *tonB*, were significantly upregulated upon PAH addition, in addition to *ompW*. This indicates that TBDTs are also involved in the transport of PAHs during the degradation process. Liang et al. analyzed the transcriptional levels of 38 TBDT family transporters in *Novosphingobium pentaromativorans* US6-1 under benzo[a]pyrene (BaP) stress and found that TBDTs may facilitate the entry of PAHs into the cell via active transport [34].

### 3.7. Heterologous Introduction of Salicylate Hydroxylase Gene

**Figure 7:**
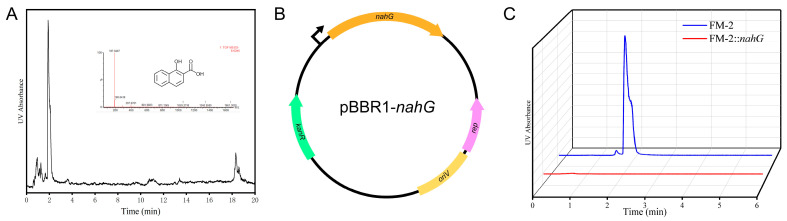
(**A**): HPLC-MS analysis results of degradation products; (**B**): Schematic diagram of plasmid construction; (**C**): Changes in the accumulation of 1-hydroxy-2-naphthoic acid before and after strain modification.

The FM-2 genome lacks genes encoding 1-hydroxy-2-naphthoate dioxygenase or salicylate hydroxylase, suggesting an inability to metabolize the phenanthrene degradation intermediate 1-hydroxy-2-naphthoic acid, which was confirmed by HPLC-MS analysis. HPLC-MS analysis revealed a distinct peak at a retention time of 1.89 min, and comparison of the mass spectrometric data confirmed the compound as 1-hydroxy-2-naphthoic acid (Figure 7A). Salicylate hydroxylase has been found to be capable of catalyzing the conversion of 1-hydroxy-2-naphthoic acid to 1,2-naphthalenediol, which has been observed to enter the naphthalene degradation pathway and ultimately be funneled into the tricarboxylic acid (TCA) cycle for complete mineralization [35]. During the metabolic process of PAHs, the degradation intermediates often exhibit greater environmental hazards than the parent PAHs due to increased bioavailability [36]. Consequently, it is imperative to minimize the accumulation of PAH degradation intermediates. To enable the metabolism of 1-hydroxy-2-naphthoic acid, the salicylate hydroxylase gene (*nahG*) from *Pseudomonas putida* PpG7 was introduced into the FM-2 strain. [37] Inspired by a report from Ouyang et al. on a series of endogenous constitutive promoters in *Burkholderia* strains for secondary metabolite production, we selected the promoter and ribosome binding site (RBS) of the 50S ribosomal protein L32 gene from the FM-2 genome to drive the expression of the *nahG* gene [38]. The *nahG* gene was inserted into the broad-host-range plasmid pBBR1 (Figure 7B). The constructed plasmid was then introduced into the FM-2 strain via electroporation, resulting in the FM-2::*nahG* strain. FM-2 and FM-2::nahG strains were cultured for three days in medium supplemented with 2 mM phenanthrene, followed by analysis using HPLC-MS. The results showed that, compared to the unmodified FM-2 strain, the FM-2::*nahG* strain completely eliminated the accumulation of the intermediate 1-hydroxy-2-naphthoic acid produced during phenanthrene degradation. This approach helps reduce secondary pollution during the biodegradation process and enhances the feasibility of microbial remediation.

## 4. Discussion

### 4.1. The PAH Degradation Capability of FM-2

FM-2 exhibits strong tolerance to various PAHs, including phenanthrene, fluorene, dibenzofuran, dibenzothiophene, and pyrene, making it a promising candidate engineering strain for PAH-contaminated remediation. FM-2 showed the highest degradation efficiency for 2 mM phenanthrene, achieving a degradation rate of 81.98%, which exceeds the degradation rate of 49.25% for 0.28 mM phenanthrene reported by Zhang et al. using *Streptomyces* sp. M-1 [6]. The degradation rate for 2 mM fluorene was found to be 68.37%, which is higher than the 66% degradation rate for 1.8 mM fluorene reported by Paul et al. using *Mucorirregularis* strain bpo1 [39]. The degradation rates for dibenzofuran and dibenzothiophene were 61.13% and 58.37%, respectively. For comparison, *Arthrobacter* sp. W1 completely degraded 0.2 mM dibenzofuran within 40 h, and the degradation rate for 0.2 mM dibenzothiophene reached 81.5% [40]. Due to factors such as the size of the active site pocket of the oxygenase and the affinity of amino acid residues, FM-2 is almost incapable of degrading pyrene. The degradation rate of *Bacillus* sp. PAH-2 on pyrene reported by Kong et al. was only 12.83% [41]. Modifying the active site pocket can expand the substrate range of oxygenases. Guo et al. engineered NarA2B2 from *Hydrogenibacillus* sp. strain N12, successfully enhancing its degradation capability toward high-molecular-weight PAHs [42].

### 4.2. Analysis of PAH Degradation Gene Clusters in FM-2

Genome sequencing can decipher the potential applications of microorganisms [43]. Through genome sequencing and transcriptomic analysis, a gene cluster involved in PAH degradation was identified in strain FM-2. Compared with some previous reports on *Burkholderia* PAH degradation gene clusters, several new degradation-related genes have been identified [4,9]. The phenanthrene degradation pathway in FM-2 was proposed. However, the absence of salicylate hydroxylase genes in the FM-2 genome suggests the possible accumulation of the intermediate 1-hydroxy-2-naphthoic acid during degradation, which is consistent with the phenomenon observed in *Marinobacter* sp. N4 [21]. Further RT-qPCR analysis confirmed that this gene cluster plays a role in the degradation of various PAHs, including phenanthrene, fluorene, dibenzofuran, and dibenzothiophene. A phylogenetic analysis was conducted on the oxygenase genes involved in PAH degradation by strain FM-2. Based on the results, the seven oxygenase genes were classified into four groups: aromatic ring-opening oxygenases, aromatic ring-hydroxylating oxygenase alpha subunits, aromatic ring-hydroxylating oxygenase beta subunits, and vicinal oxygen chelate (VOC) proteins. The sequences of oxygenases within the same group were relatively conserved. Finally, the transporter gene ompW, located downstream of the gene cluster, was investigated. Its structure was predicted using AlphaFold3, and the potential channel through which PAHs molecules might pass within OmpW was inferred.

### 4.3. Optimization of the PAH Degradation Pathway

HPLC-MS results confirmed the significant accumulation of the intermediate 1-hydroxy-2-naphthoic acid during phenanthrene degradation by FM-2. Some studies have attempted to remove this intermediate. Wang et al. removed 1-hydroxy-2-naphthoic acid and improved phenanthrene degradation efficiency by co-culturing *Marinobacter* sp. N4 with *Halomonas* sp. G29 [21]. Zhang et al. constructed an artificial consortium by introducing phenanthrene degradation genes from different species into *E. coli* BL21 strains to achieve phenanthrene mineralization [44]. However, there has been no report on directly introducing a heterologous salicylate hydroxylase into PAH-degrading bacteria. In this study, the salicylate hydroxylase from *Pseudomonas putida* PpG7 was combined with an endogenous promoter from FM-2 and introduced into FM-2 via the pBBR1 plasmid. The results showed that the engineered strain FM-2::*nahG* completely eliminated the accumulation of 1-hydroxy-2-naphthoic acid, achieving complete mineralization of phenanthrene. This approach helps reduce secondary pollution during the microbial remediation process and enhances the feasibility of microbial remediation.

## 5. Conclusions

This study investigated the degradation capability of *Burkholderia* sp. FM-2 towards various PAHs. The results demonstrated that FM-2 exhibited good tolerance and degradation ability towards multiple PAHs, including phenanthrene, fluorene, dibenzofuran, and dibenzothiophene. Through genome sequencing and transcriptomic analysis, a gene cluster involved in PAH degradation was identified in strain FM-2, which contains five dioxygenase genes and two monooxygenase genes. The oxygenase genes and transporter protein genes within the PAH degradation gene cluster were analyzed through RT-qPCR and bioinformatics. Finally, to eliminate the accumulation of the intermediate 1-hydroxy-2-naphthoic acid during phenanthrene degradation by FM-2, the strain was genetically modified. This study is the first report on eliminating the intermediate 1-hydroxy-2-naphthoic acid by introducing a heterologous salicylate hydroxylase gene (*nahG*) into the degrading bacterium. The *Burkholderia* sp. FM-2 and FM-2::nahsG constructed in this study exhibits promising potential for application in the remediation of environmental pollution. This study can also provide guidance for the further engineering and optimization of PAH-degrading microorganisms.

## Data Availability

The original contributions presented in the study are included in the article/Supplementary Material, further inquiries can be directed to the corresponding authors.

## Supplementary Materials

The following supporting information can be downloaded at: https://www.mdpi.com/article/10.3390/microorganisms13092079/s1, Section S1: nahG sequence; Figure S1: Color changes during FM-2 cultivation (from left to right: phenanthrene, fluorene, pyrene, dibenzofuran, dibenzothiophene); Figure S2: Surface hydrophobicity analysis of the OmpW protein (A: intracellular side; B: extracellular side); Table S1: Primers used in RT-qPCR.
