# Supplementary material for: Study and Modification of the Polycyclic Aromatic Hydrocarbon Degradation Gene Cluster in Burkholderia sp. FM-2
Source: Microorganisms. 2025 Sep 6;13(9):2079. doi: 10.3390/microorganisms13092079 (PMC12472010; doi:10.3390/microorganisms13092079)
Supplement: Supplementary file 1 [file microorganisms-13-02079-s001.zip › microorganisms-3834376-supplementary.pdf]

[Supplementary information]

Study and Modification of the Polycyclic Aromatic Hydrocarbon Degradation Gene Cluster in *Burkholderia* sp. FM-2

Supplementary Sections

Section S1 | *nahG* sequence.

AAGCTTTGAACAGTTGCATGGGAGCGCGAAGCGGGCGCATTGGCCATTCGGTCGATGGCGGACAC
CGGGTCGGGCTGTGTTAGAATTCGGAAAATTTTTAGGAGTTAGTCATGAAGAACAACAAGCTCGG
CCTGCGCATCGGCATTGTGGGCGGCGGTATCTCGGGCGTGGCGCTGGCGCTGGAACTGTGCCGCT
ACTCGCACATCCAAGTGCAGCTGTTCGAAGCGGCCCCGGCGTTTGGCGAAGTGGGTGCCGGCGTC
TCGTTTGGCCCGAACGCGGTGCGCGCCATTGTGGGCCTCGGCCTGGGCGAAGCGTACCTCCAAGT
GGCGGACCGCACGTCGGAACCGTGGGAAGACGTGTGGTTCGAATGGCGCCGCGGTTCGGACGCGT
CGTACCTGGGCGCCACGATTGCGCCGGGTGTGGGCCAATCGTCGGTGCACCGCGCGGACTTCATC
GACGCGCTGGTGACGCACCTGCCGGAAGGCATCGCGCAGTTCGGCAAGCGCGCGACGCAAGTGGA
ACAGCAAGGCGGCGAAGTGCAAGTGCTGTTCACGGACGGCACGGAATACCGCTGCGACCTGCTGA
TTGGCGCGGACGGCATCAAAAGCGCGCTCCGGAGCCATGTGCTCGAAGGTCAAGGCCTGGCCCCG
CAAGTGCCCCGGTTCTCGGGCACGTGTGCGTACCGCGGCATGGTGGACTCGCTGCACCTCCGCGA
AGCGTATCGCGCGCACGGCATCGACGAACACCTGGTGGACGTGCCGCAGATGTACCTGGGCCTGG
ACGGCCACATCCTGACGTTCCCGGTGCGCAACGGCGGCATCATCAACGTGGTGGCGTTCATCAGC
GACCGCAGCGAACCGAAACCGACCTGGCCCGCCGACGCCCCCTGGGTGCGCGAGGCGTCGCAGCG
GGAAATGCTCGATGCGTTCGCGGGTTGGGGCGATGCCGCCCGCGCCCTGCTGGAATGCATCCCGG
CCCCGACGCTCTGGGCGCTGCACGATCTGGCGGAACTCCCGGGTTACGTGCATGGCCGCGTCGTC
CTGATTGGTGATGCGGCGCACGCGATGCTGCCGCACCAAGGCGCGGGTGCGGGTCAAGGCCTCGA
AGATGCGTACTTCCTGGCGCGGCTCCTCGGTGATACCCAAGCGGACGCGGGCAACCTCGCGGAAC
TGCTGGAAGCCTACGATGATCTCCGGCGCCCGCGCGCCTGTCGCGTGCAGCAGACGTCGTGGGAA
ACGGGCGAACTGTACGAACTGCGCGACCCGGTGGTGGGCGCGAACGAACAGCTGCTGGGCGAAAA
CCTGGCGACGCGCTTCGACTGGCTGTGGAATCACGATCTGGATACCGATCTCGCCGAGGCCCGCG
CCCGCCTCGGTTGGGAACACGGCGGTGGCGGCGCGCTGCGCCAAGGCTGAGGATCC

5'HindIII highlighted in Yellow
3'BamHI highlighted in Cyan
Promoter and RBS highlighted in Green

List of Figures and Tables

Figure S1. Color changes during FM-2 cultivation (from left to right: phenanthrene, fluorene, pyrene, dibenzofuran, dibenzothiophene).

Figure S2 Surface hydrophobicity analysis of the OmpW protein (A: intracellular side; B: extracellular side.

Table S1 Primers used in RT-qPCR.

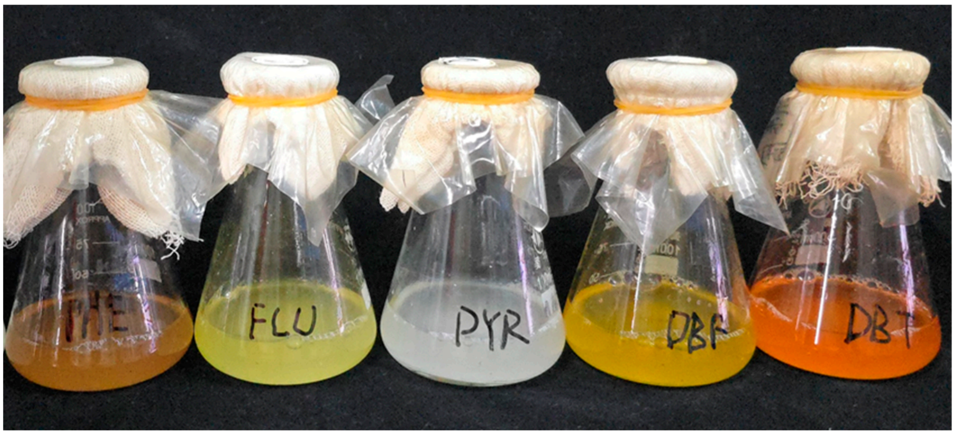

Figure S1 Color changes during FM-2 cultivation (from left to right: phenanthrene, fluorene, pyrene, dibenzofuran, dibenzothiophene)

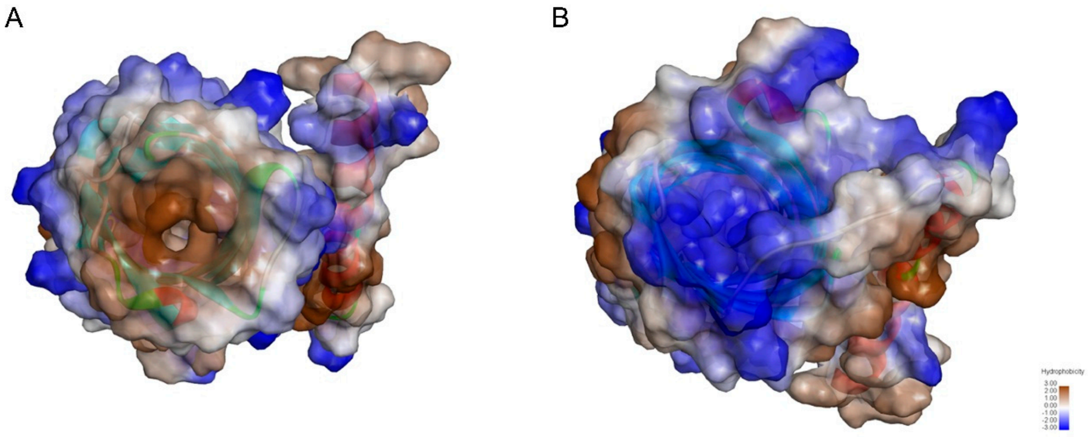

Figure S2 Surface hydrophobicity analysis of the OmpW protein (A: intracellular side; B: extracellular side)

Table S1 Primers used in RT-qPCR

Primer name	Sequence
16sRNA-F	TCAGCAGGATTCCATACATGTC
16sRNA-R	ACAGGATTAGATACCCTGGTAGTC
nagI-F	TGCTCATTTCGGATACAAGCTG
nagI-R	CGTTTGCATCAAAGGAGAACG
phnAc-F	GGTGCAAAGGACGTTTGG
phnAc-R	GCAGAATCACCGACGATACC
phnAd-F	GAAGCCCGACTGATGGATAC
phnAd-R	CGAATGCCTAGCTGTCTGTG
nagG-F	ACTGGTGTTATGTTGGTCTGG
nagG-R	TCAGCGAGTAGTTCCATTGATG
nagH-F	TGAGATGGTTGACTTCGATACG
nagH-R	AGTACGGGTCGTGAAACAG
phnCa-F	AGCATCTACACCGTAGAACG
phnCa-R	CGTCTTTAAAGACGAGTGTTCATCC
phnCb-F	ACTACAGGTCGACACTGTAGTG
phnCb-R	GATCGAGAACGAGCGACTTC
ompW-F	TCGGCAAAGTGGAATATGCTC
ompW-R	CAAACAGTCCAACATGCTTCG
tonB-F	CGTTTCGCACATCGACTG
tonB-R	GTTCTCGAGATAGGGTTTGCAG
exbB-F	ATGTGATGCGCCTGAACc
exbB-R	GACACGTCCATACGATCGTG
exbD-F	CAAGCAGTCAGAAGGAAGACAC
exbD-R	TTCTCATACGCGACCTTGC
